# Conserved intracellular virulence architecture and focal genomic diversification in sub-Saharan African *Brucella melitensis*

**DOI:** 10.1128/spectrum.01703-26

**Published:** 2026-08-19

**Authors:** Samweli Y. Bahati, Eliezer Brown Mwakalapa, Henry G. Mung'ong'o, Abdalah Makaranga, Benson Kidenya, Reuben S. Maghembe

**Affiliations:** 1 Department of Omics & Computational Biology, AfroBiomics Co. Ltd., Dar es Salaam, Tanzania; 2 Department of Microbiology and Parasitology, Faculty of Medicine, St. Francis University College of Health and Allied Sciences 518753, Ifakara, Tanzania; 3 Department of Natural Sciences, Mbeya University of Science and Technology 196572, Mbeya, Tanzania; 4 Institute of Accountancy Arusha (IAA) 108067, Arusha, Tanzania; 5 Department of Agriculture, Earth and Environmental Sciences, Faculty of Science, Mwenge Catholic University (MWECAU) 322280, Moshi, Tanzania; 6 Department of Biochemistry and Molecular Biology, Weill Bugando School of Medicine, Catholic University of Health and Allied Sciences 361475 , Mwanza, Tanzania; Yale School of Medicine, New Haven, Connecticut, USA

**Keywords:** *Brucella melitensis*, brucellosis, comparative genomics, whole-genome sequencing, phylogenomics, pangenome, virulence factors, antimicrobial resistance, mobile genetic elements, zoonotic genomic surveillance, sub-Saharan Africa

## Abstract

*Brucella melitensis* has a highly conserved genome, but the distribution of core-genome, gene-content, virulence-associated, mobile-element-associated, and antimicrobial-resistance-relevant variation among available sub-Saharan African genomes has not been examined in an integrated regional analysis. We analyzed 51 curated *B. melitensis* genomes from human and animal hosts using core-genome phylogenomics, pangenome reconstruction, virulence profiling, mobile genetic element (MGE) analysis, and mutation-based screening of antimicrobial resistance (AMR)-associated loci. A phylogeny reconstructed from 6,060 shared SNP sites resolved a dominant ST12-associated lineage, together with ST7, ST8, ST42, and novel sequence type branches. Individual genomes differed from the reference by 1,677–2,579 SNPs. The pangenome comprised 3,457 gene families, including 3,049 persistent families, indicating strong genome conservation, and limited accessory expansion. Virulence profiling identified 66 VFDB-associated genes; 48 genomes carried all 66, and the remaining three retained more than 98% of the virulence repertoire. Conserved determinants included the VirB type IV secretion system, lipopolysaccharide biosynthesis, intracellular survival pathways, and stress-response functions. MGE-associated variation was restricted to a small number of regions dominated by transposases and insertion-sequence-associated proteins. A GspF-domain-containing secretion-associated locus was detected only in BM2, although no complete type II secretion system gene cluster was identified. Recurrent substitutions occurred in AMR-relevant chromosomal loci, including *rpoB*, *gyrA*, *gyrB*, *parC*, *parE*, *folA*, *folP*, *bepCDEFG,* and *mprF*, but none corresponded to validated resistance-conferring alleles. The available regional genomes therefore comprise multiple phylogenetic lineages within a strongly conserved gene and intracellular virulence framework, with diversity concentrated in core-genome SNPs and localized genomic regions.

**IMPORTANCE:**

*Brucella melitensis* is a major zoonotic pathogen at the livestock-human interface, but genome-resolved evidence from sub-Saharan Africa remains limited. This study curates available regional genomes and shows that the population is dominated by a conserved intracellular virulence backbone, strong core-genome conservation, and focal genomic diversification rather than by extensive accessory-genome expansion. By integrating phylogenomics, pangenome analysis, virulence profiling, mobile-element characterization, and mutation screening of antimicrobial-resistance-associated loci, the work provides a regional framework for One Health genomic surveillance and identifies candidate loci requiring phenotype-linked validation.

## INTRODUCTION

*Brucella melitensis* is the leading cause of human brucellosis and is primarily maintained in sheep and goats, although cattle and camels can also become infected and contribute to transmission in some endemic livestock systems (1–3). In sub-Saharan Africa, incomplete case detection, limited laboratory confirmation, and fragmented human and animal surveillance constrain estimates of disease burden and the characterization of circulating *Brucella* lineages (4). Whole-genome analysis provides greater discriminatory resolution than classical typing for distinguishing closely related isolates, resolving phylogenetic lineages, and supporting outbreak source tracing when genomic relationships are evaluated with epidemiological metadata (5).

*Brucella melitensis* has a highly conserved genome architecture and a predominantly intracellular pathogenic program. Its virulence repertoire includes the VirB type IV secretion system and its effectors, smooth lipopolysaccharide and O-antigen biosynthesis, cyclic β-1,2-glucan, adhesins, urease and acid-resistance functions, oxidative and nitrosative stress defenses, and pathways involved in Brucella-containing-vacuole trafficking and persistence within macrophages (6, 7). Pangenome analysis provides a framework for quantifying persistent and accessory gene content and identifying strain-restricted genes and regions of genomic plasticity within this conserved genomic background. Genomic studies have also reported lineage-associated variation in mobile elements, accessory genes, and chromosomal loci associated with rifampicin, fluoroquinolone, and folate-pathway susceptibility, although the phenotypic significance of many reported mutations remains unresolved (8, 9).

Despite growing sequence availability, sub-Saharan African *B. melitensis* genomes remain sparsely and unevenly represented across countries and hosts, limiting assessment of lineage distribution and genomic diversity within the available regional collection. In this study, we examined whether genomic diversity in genomes from human and animal hosts was concentrated in core-genome SNPs and focal accessory regions against a conserved intracellular virulence background. We addressed this question by integrating core-genome phylogenomics, pangenome reconstruction, virulence profiling, mobile genetic element analysis, and mutation-based screening of antimicrobial resistance-associated loci.

## MATERIALS AND METHODS

### Genome data set collection and metadata curation

A total of 53 publicly available *B. melitensis* whole-genome data sets associated with sub-Saharan Africa were retrieved from the National Center for Biotechnology Information (NCBI; https://www.ncbi.nlm.nih.gov/) database. Metadata, including host source, specimen type, collection year, and geographical origin, were curated from associated BioSample, GenBank, and Sequence Read Archive (SRA; https://www.ncbi.nlm.nih.gov/sra) records.

### Read preprocessing, genome assembly, and quality assessment

Raw sequencing reads were quality filtered and adapter trimmed using fastp v0.24.0 (10). High-quality reads were assembled *de novo* using SPAdes genome assembler v4.2.0 (11). Assembly quality metrics, including contig statistics and assembly completeness, were assessed using QUAST v5.3.0 (https://quast.sourceforge.net/docs/manual.html).

### Contamination screening and species confirmation

Assemblies were screened for contamination before comparative analysis. Taxonomic validation was performed using Pathogenwatch (https://pathogen.watch/en) together with ContEst16S (12), which was used to identify discordant non-*Brucella* fragments within assembled genomes. Two genomes containing contaminating sequences assigned to non-*Brucella* taxa were excluded, resulting in a final curated data set of 51 genomes.

### Genome annotation

Genome annotation was performed using Bakta v1.11.0 (13). Resulting annotations were used for downstream extraction and classification of virulence-associated genes, resistance-associated loci, mobile genetic element (MGE)-associated features, and other functional categories.

### Sequence typing and core-genome phylogenomics

*In silico* sequence typing was performed using the *Brucella* MLST framework integrated within Pathogenwatch (14). Core-genome SNP calling was conducted using Snippy v4.6.0 (15). Shared SNP positions across all genomes were extracted to generate a high-quality core alignment comprising 6,060 SNP sites. Maximum-likelihood phylogenetic reconstruction was performed using IQ-TREE multicore v2.4.0 (16).

### SNP annotation and mutation analysis

Functional annotation of SNPs was performed using SnpEff v5.2 (17). Variants located within genes associated with antimicrobial susceptibility, efflux systems, membrane biology, and stress adaptation were extracted for downstream analysis. Genes examined included *rpoB*, *gyrA*, *gyrB*, *parC*, *parE*, *folA*, *folP*, *bepC*, *bepE*, *bepF*, and *mprF*.

### Pangenome analysis

Pangenome reconstruction was performed using PPanGGOLiN v2.2.2 (https://anaconda.org/channels/bioconda/packages/ppanggolin/overview). Gene families were partitioned into persistent, shell, and cloud components according to genome frequency distribution. Regions of genomic plasticity (RGPs), spots, and modules were identified within the partitioned pangenome graph.

### Virulence factor profiling and identification of putative resistance-associated loci

Virulence-associated genes were identified using the Virulence Factor Database (VFDB) (18). Virulence loci were classified into functional categories. Resistance-associated loci were identified through a literature-guided curation strategy based on Bakta annotations.

### Mobile genetic element analysis

MGE-associated loci, including transposases, insertion sequence-associated proteins, recombinases, and integrases, were extracted from Bakta annotations. These loci were re-annotated together with neighboring genomic regions to characterize associated passenger and mobility-related genes.

### Secretion-associated protein analysis

A secretion-associated locus annotated as a Type II secretion system protein GspF domain-containing protein was identified in one genome. Homologous sequences were retrieved using similarity-based searches and aligned for phylogenetic reconstruction to assess evolutionary relationships with related secretion-associated proteins using BLASTp (https://blast.ncbi.nlm.nih.gov/Blast.cgi?PROGRAM=blastp&PAGE_TYPE=BlastSearch).

### Data visualization and statistical analysis

Phylogenetic trees, pangenome summaries, virulence distributions, MGE classifications, and mutation profiles were visualized using iTOL v7 (19), Linux-based workflows, and R-based graphical analyses. Descriptive statistics were performed in R.

## RESULTS

### A curated sub-Saharan African genome panel captures human and animal *B. melitensis* diversity

We compiled 53 *Brucella melitensis* genomes from sub-Saharan Africa to construct a region-focused comparative data set spanning both human and animal hosts. Manual curation identified two assemblies with clear evidence of contamination, each containing non-*Brucella* fragments assignable to other bacterial taxa. One genome contained fragments matching *Streptococcus porcorum* and *Pasteurella multocida* subsp. *septica*, whereas the second contained a fragment matching *Pasteurella multocida* subsp. *multocida*. Both were removed, leaving a final high-confidence data set of 51 genomes for all downstream analyses. More details are provided in Data S1.

The curated panel encompassed isolates from multiple host backgrounds, including humans, goats, sheep, and cattle, supporting a cross-host view of *B. melitensis* diversity in the region. Geographic metadata showed that the collection was centered on Ethiopia, but also included genomes from Somalia, Tanzania, Nigeria, Zimbabwe, South Africa, and Sudan, thus capturing regional breadth across eastern, western, and southern Africa. Collection dates ranged from historical to recent isolates. More details are provided in Data S2.

### Core-genome phylogenomics and sequence typing reveal structured diversification within the sub-Saharan African *B. melitensis*

**Fig 1:**
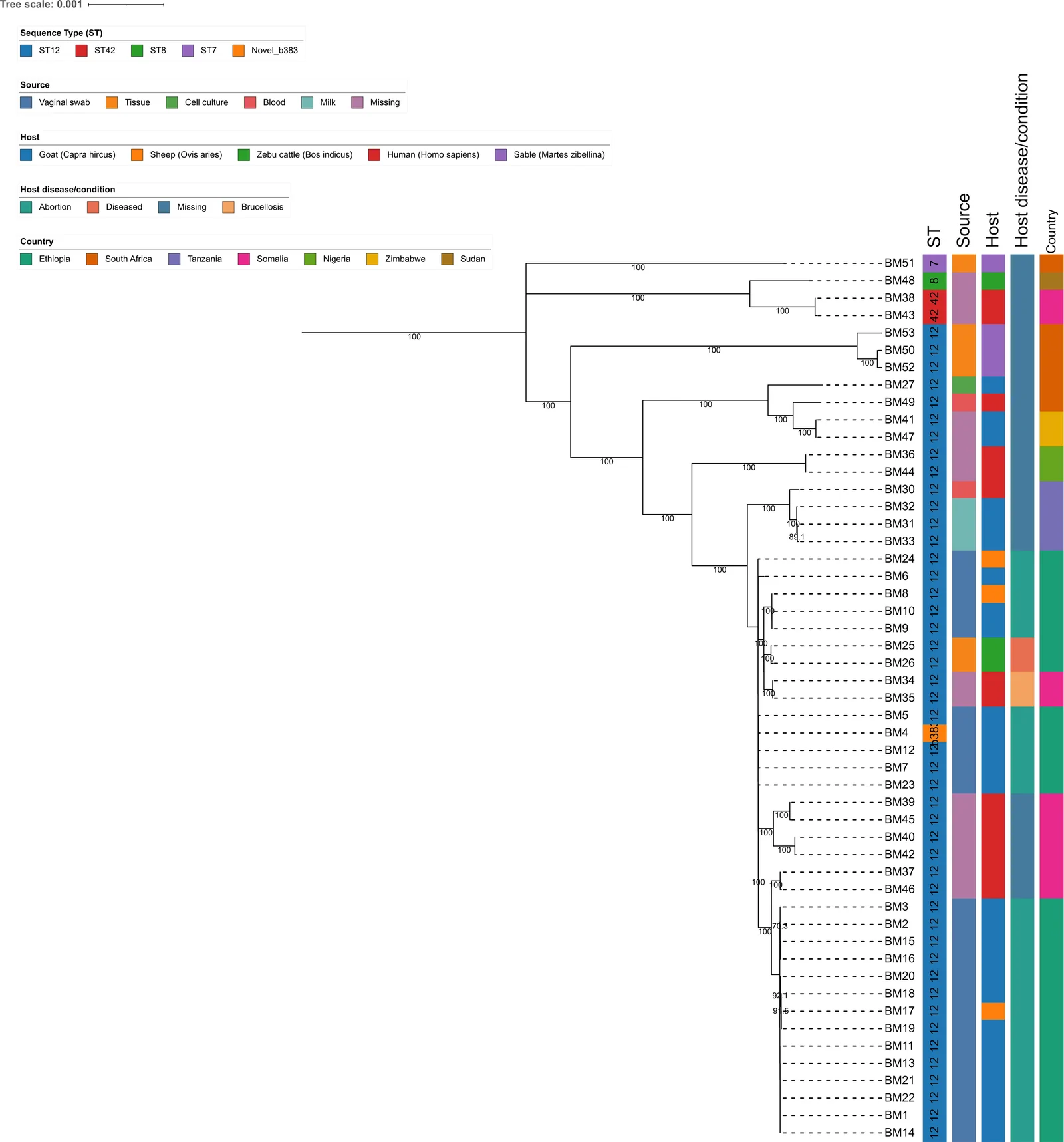
Population structure and metadata distribution of *Brucella melitensis* isolates from Sub-Saharan Africa. Maximum-likelihood phylogeny of 51 *B. melitensis* genomes reconstructed from core-genome single-nucleotide polymorphisms (SNPs), illustrating the genetic relationships among isolates recovered from multiple hosts, sample sources, clinical conditions, and geographic regions across Sub-Saharan Africa. Colored annotation strips adjacent to the phylogeny indicate sequence type (ST), source of isolation, host species, host disease/clinical condition, and country of origin.

Phylogenetic reconstruction based on a high-quality core-genome alignment of 6,060 SNP sites resolved distinct phylogenetic lineages within the 51-genome sub-Saharan African panel (Fig. 1). Across the data set, individual genomes differed from the reference by 1,677–2,579 SNPs.

The majority of genomes formed a single large clade assigned to ST12, comprising isolates BM1–BM37, BM39–BM47, and BM49–BM53 (Fig. 1). Within this clade, several closely related pairs or subclusters were present, including BM9/BM10, BM35/BM34, and BM44/BM36, all within ST12. Outside this dominant clade, distinct lineages were observed. BM48 formed an independent branch and was assigned to ST8. A separate lineage comprised BM51, assigned to ST7. Two genomes, BM38 and BM43, formed a distinct clade assigned to ST42. A single genome, BM4, was assigned to a novel sequence type (Novel_b383) and occupied an individual position in the tree.

Hosts were distributed across the phylogeny. The large ST12 clade included isolates from goats (*Capra hircus*), sheep (*Ovis aries*), zebu cattle (*Bos indicus*), and humans (*Homo sapiens*). The ST8 lineage, represented by BM48, was recovered from zebu cattle (*Bos indicus*), whereas the ST7 lineage, including BM51 and the associated BM50–BM53 genomes, was recovered from sable (*Martes zibellina*). The ST42 clade, comprising BM38 and BM43, consisted of human (*Homo sapiens*) isolates (Fig. 1).

### Conserved core with limited accessory expansion dominates the pangenome

Across the 51 genomes, the pangenome comprised 158,443 genes grouped into 3,457 gene families. Of these, 3,049 families (88.2%) were assigned to the persistent partition, whereas only 63 families (1.8%) fell within the shell and 345 families (10.0%) within the cloud. Persistent families were present in 92–100% of genomes, with a mean genome frequency of 1.0 and a standard deviation of 0.01. Shell families occurred in 27–90% of genomes (mean 0.65, SD 0.19), whereas cloud families were restricted to 2–29% of genomes (mean 0.04, SD 0.05). The partitioning, therefore, resolved a pangenome overwhelmingly dominated by the persistent fraction, with comparatively limited shell and cloud components.

**Fig 2:**
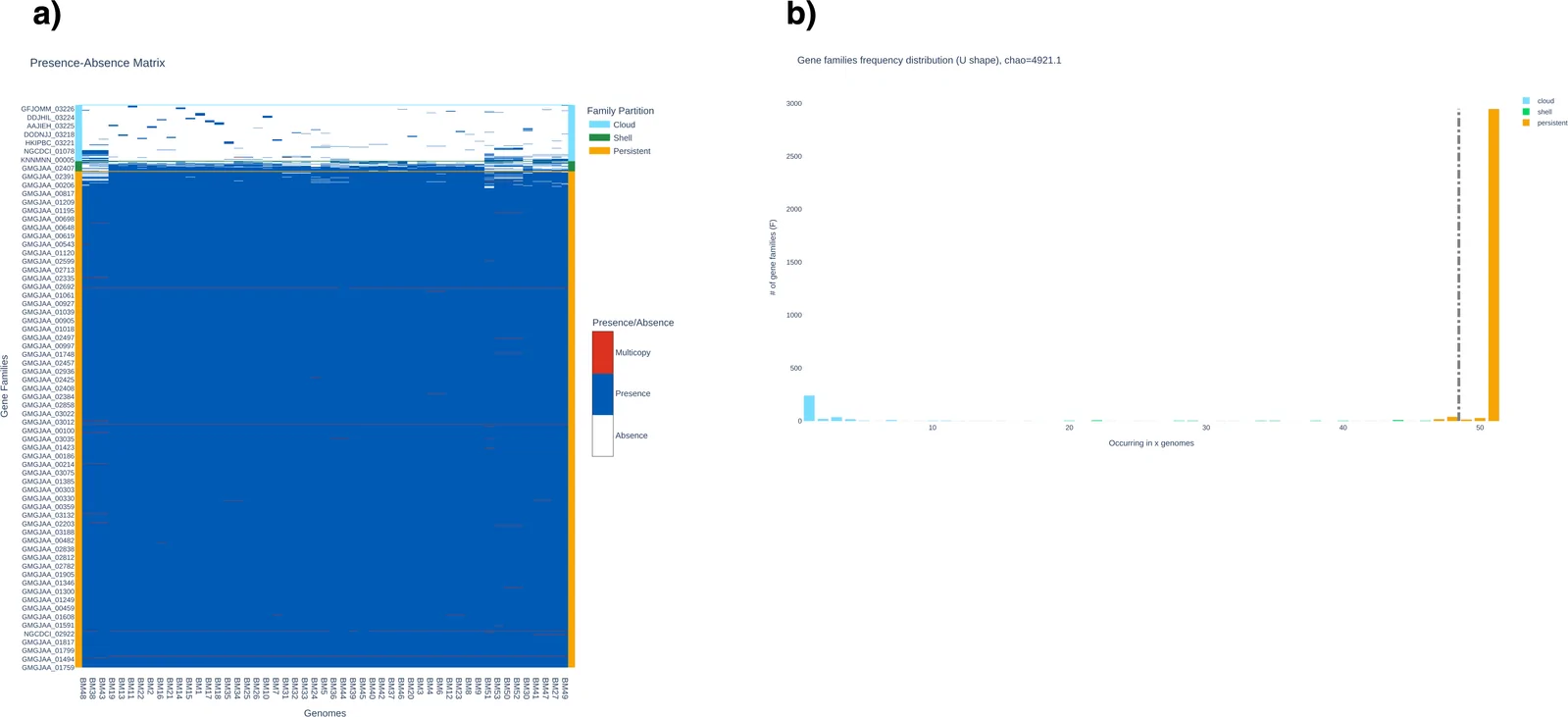
Pangenome architecture and gene family distribution of *Brucella melitensis* genomes from Sub-Saharan Africa. (**a**) Presence–absence matrix showing the distribution of gene families across 51 *B. melitensis* genomes. Rows represent gene families and columns represent individual genomes. Gene families were partitioned into persistent (core), shell, and cloud components according to their prevalence across the data set. Blue indicates gene presence, red indicates multicopy occurrence, and white indicates absence. (**b**) Frequency distribution of gene families across the analyzed genomes, illustrating the characteristic U-shaped pangenome profile.

The frequency distribution of gene families showed a pronounced U-shaped profile (Fig. 2), with a large accumulation of families at the persistent end and a smaller low-frequency tail at the cloud end. At the high-frequency extreme, families present in all 51 genomes accounted for 2,948 persistent families, with additional persistent counts at frequencies 50 (28 families), 49 (16)**,** 48 (40), and 47 (17). At the low-frequency extreme, the cloud fraction was concentrated in rare families, including 242 families present in a single genome, 20 in two genomes, 37 in three genomes, and 17 in four genomes. The corresponding richness estimate from the U-shaped distribution was Chao = 4921.1.

Genome plasticity was limited at the pangenome-graph level. Only three regions of genomic plasticity (RGPs) and one spot were detected, together with 2 modules comprising nine families in total; all module-associated families belonged to the cloud partition (100% cloud; 0% persistent; 0% shell).

**Fig 3:**
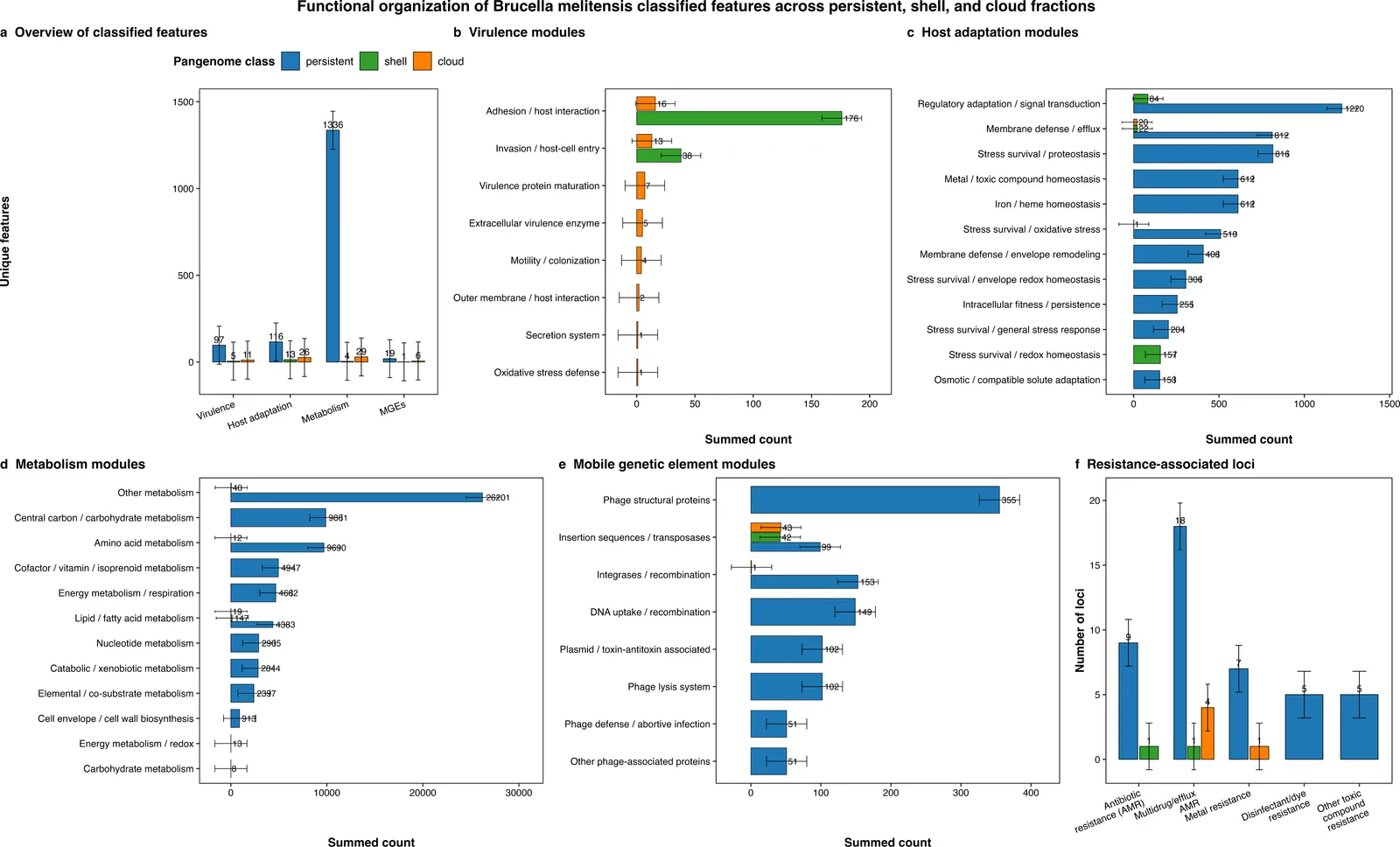
Functional organization of classified genomic features across the *Brucella melitensis* pangenome. Comprehensive functional characterization of persistent, shell, and cloud gene fractions identified across 51 *B. melitensis* genomes. (**a**) Overview of classified genomic features distributed across virulence, host adaptation, metabolic, and mobile genetic element (MGE)-associated categories within the pangenome structure. (**b**) Distribution of virulence-associated modules. (**c**) Host adaptation modules associated with environmental sensing, membrane defense, stress survival, intracellular persistence, iron/heme homeostasis, osmotic adaptation, and regulatory signal transduction. (**d**) Metabolic organization of the pangenome, demonstrating strong enrichment of central carbon metabolism, amino acid metabolism, lipid/fatty acid metabolism, respiration, and cofactor biosynthesis pathways within the persistent genome fraction, consistent with metabolic conservation across isolates. (**e**) Distribution of mobile genetic element-associated modules, including phage structural proteins, insertion sequences/transposases, integrases, recombination systems, toxin–antitoxin-associated loci, and phage defense systems. (**f**) Resistance-associated loci identified across the genomes, including antibiotic resistance, multidrug/efflux-associated systems, metal resistance determinants, disinfectant/dye resistance genes, and other toxic compound resistance loci.

Classification of curated biological features followed the same overall architecture (Fig. 3). In the overview, the persistent partition contained the largest counts for virulence (97), host adaptation (116), metabolism (1,336), and mobile genetic element-related features (26), whereas shell and cloud counts remained smaller across all four categories.

### Virulence genes define a stable intracellular pathogenic backbone

**Fig 4:**
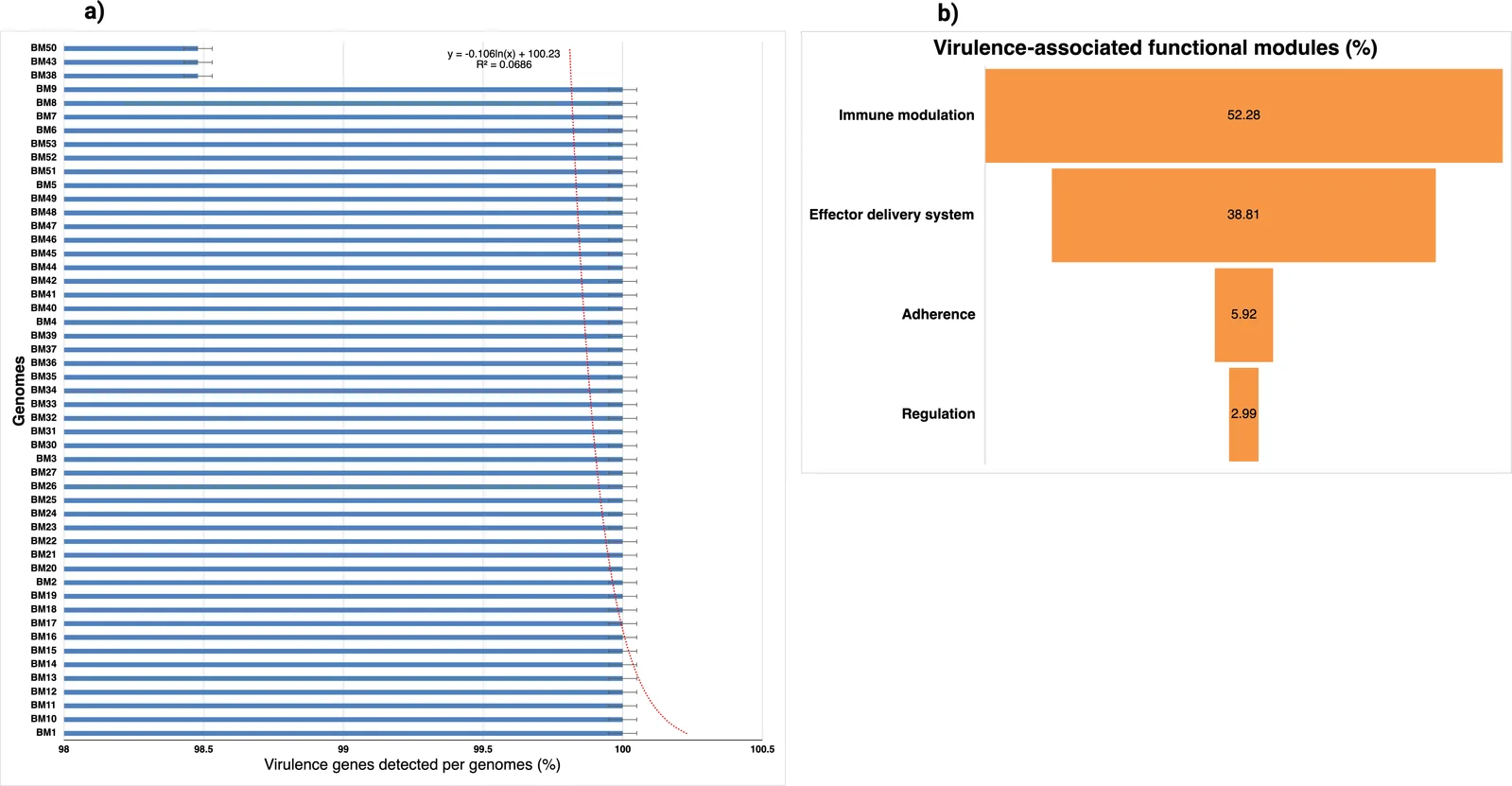
Virulence gene conservation and distribution across *Brucella melitensis* genomes. (**a**) Genome-wide prevalence of virulence-associated genes across 51 *B. melitensis* isolates. (**b**) Distribution of virulence-associated functional modules in *B. melitensis.* The complete genome-by-gene heatmap and associated metadata annotations are presented in Fig. S1.

VFDB profiling detected 66 unique virulence-associated genes across the 51 genomes. Genome-level recovery was uniformly high: 48 genomes carried all 66 genes, while three genomes each carried 65 genes (98.48%) (Fig. 4; Fig. S1). Across ranked genomes, detection ranged from 98.48 to 100.00%, with a fitted curve of *y* = −0.106 ln(*x*) + 100.23 and *R*² = 0.0686. The only variably detected VFDB genes were virB10 and bmaC, present in 50/51 and 49/51 genomes, respectively.

The VFDB repertoire covered the principal *Brucella* virulence systems, including the VirB type IV secretion system, type IV secretion effectors, LPS/O-antigen biosynthesis, outer-membrane and surface antigen functions, cyclic β−1,2-glucan, urease/acid-resistance genes, oxidative and nitrosative stress genes, respiratory/microaerobic survival genes, and DNA repair-associated virulence functions. All four VFDB module classes were detected across the full genome set: immune modulation, effector delivery system, adherence, and regulation.

At the module level, the VFDB profile was concentrated in two dominant classes. Immune modulation accounted for 52.28%, followed by the effector delivery system at 38.81%. Adherence and regulation contributed 5.92% and 2.99%, respectively (Fig. 4).

Pangenome-linked classification placed most virulence-associated features in the persistent fraction, with 97 persistent, five shell, and 11 cloud virulence features. Within the classified virulence panel, the largest absolute module counts were adhesion/host interaction (176) and invasion/host-cell entry (38), followed by virulence protein maturation (16), extracellular virulence enzyme (13), motility/colonization (5), outer membrane/host interaction (4), secretion system (2), and oxidative stress defense (1) (Fig. 3).

### Mobile-associated regions mark focal zones of plasticity

**Fig 5:**
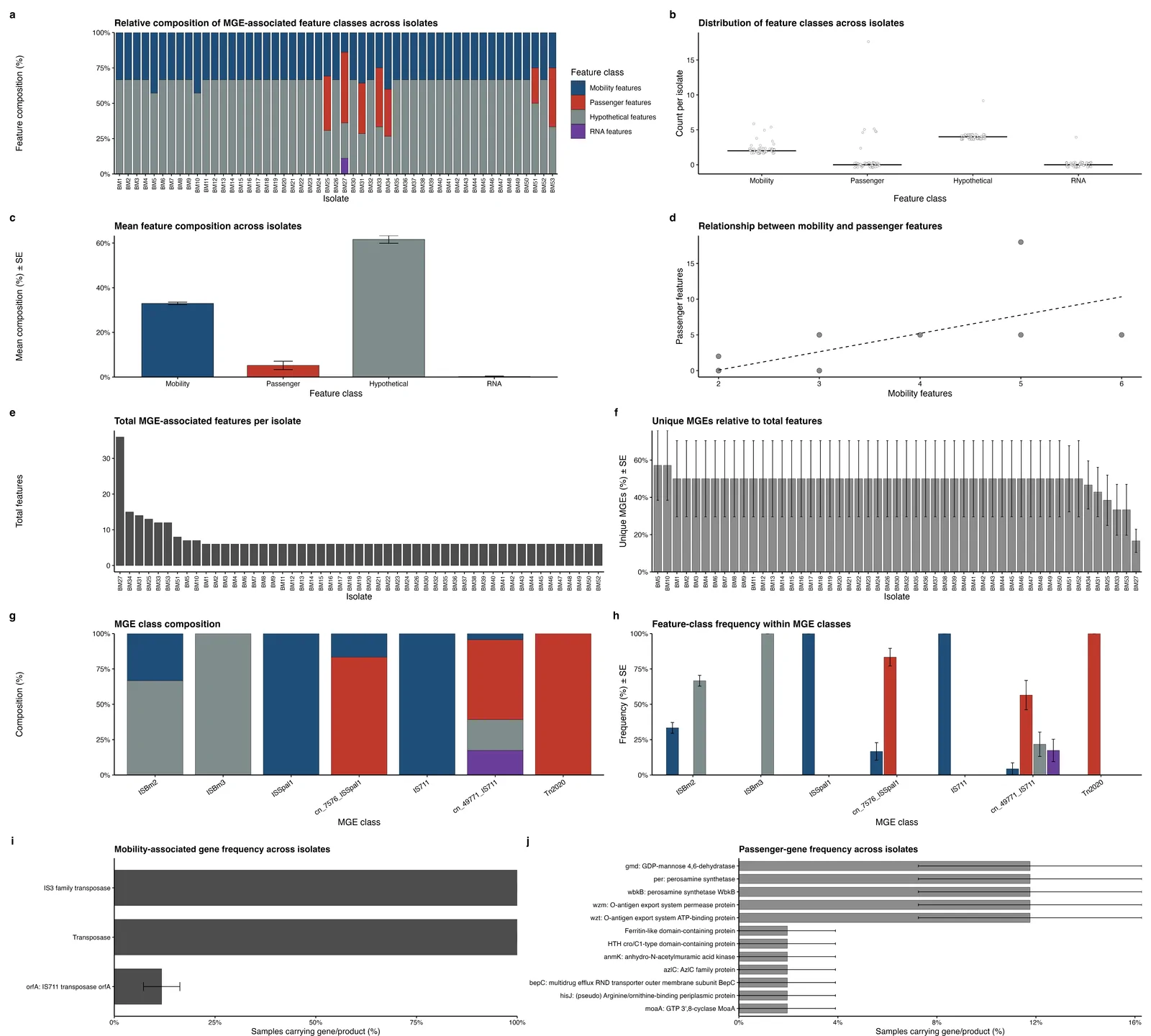
Comparative landscape of mobile genetic element (MGE)-associated features across *Brucella melitensis* genomes. Comprehensive characterization of MGE-associated feature classes identified across the analyzed *B. melitensis* isolates. (**a**) Relative composition of MGE-associated feature classes across individual isolates, including mobility-associated features, passenger genes, hypothetical features, and RNA-associated elements. (**b**) Distribution of feature-class counts across isolates. (**c**) Mean composition of MGE-associated feature classes across the data set expressed as percentage ± standard error. (**d**) Correlation between mobility-associated and passenger-associated features across isolates. (**e**) Total number of MGE-associated features detected per isolate. (**f**) Relative proportion of unique MGE-associated features across isolates. (**g**) Composition of major MGE classes identified across the data set, including insertion sequence (IS) families and transposon-associated elements. (**h**) Frequency distribution of feature classes within each MGE category. (**i**) Prevalence of mobility-associated genes across isolates. (**j**) Distribution of passenger-associated genes across isolates, including loci linked to membrane transport, ferritin-like proteins, polysaccharide biosynthesis, stress adaptation, and metabolic functions.

MGE-associated features were unevenly distributed across the 51 genomes. Most isolates carried a small, compact MGE feature set, whereas a limited number of genomes showed expanded MGE-associated content. One isolate carried the largest burden, followed by smaller peaks in six isolates (Fig. 5).

Across isolates, the MGE feature set was dominated by hypothetical proteins and mobility genes. Mean feature composition was approximately 62% hypothetical, 33% mobility-associated, 5% passenger-associated, and near-zero RNA-associated features. Passenger features were restricted to a small subset of genomes and increased with mobility feature counts at the isolate level (Fig. 5).

At the class level, the data set contained recurrent mobility elements, including IS3 family transposase and transposase, both present across the full genome panel. A third mobility feature, orfA (IS711 transposase orfA), was present in a smaller subset of genomes. MGE classes showed distinct compositions: IS711/ISSpn1-like classes were mobility-dominated, ISBm3 was hypothetical-dominated, and cn_7516_ISKpn1 and TnAs2-like classes were passenger-dominated.

Passenger genes were less frequent than mobility genes. The most recurrent passenger features included gmd, per, wbkB, wzm, and wzt, each detected in a minority of genomes, followed by lower-frequency genes including ferritin-like, HTH-domain, ankyrin-repeat, acyl-carrier, efflux-related, and arginine-rich protein (Fig. 5). More details are provided in the Data S3.

In the pangenome-linked classification, MGE-associated features were distributed across 26 persistent, four shell, and 19 cloud features, with insertion sequences/transposases, integrases/recombination proteins, DNA uptake/recombination functions, phage structural proteins, and toxin–antitoxin-associated features forming the principal annotated MGE modules (Fig. 3).

### Conserved efflux and detoxification loci dominate the resistance-associated annotation profile

Literature-guided screening of Bakta annotations identified a compact set of putative resistance-associated loci across the 51 genomes (Table 1). The primary curated AMR set comprised eight genes detected in all genomes (51/51; 100%): *norM*, *fosX*, *macB*, *bepC*, *bepD*, *bepE*, *bepF*, and *bepG*. These loci encoded predicted multidrug/toxic-compound efflux, fosfomycin hydrolase activity, macrolide export, and *Brucella* RND efflux components.

**TABLE 1:** Antimicrobial resistance-associated genes identified in *Brucella melitensis* genomes based on Bakta annotation^*a*^

Gene	Predicted product	No. isolates (*n* = 51)	Prevalence (%)
*norM*	NorM multidrug and toxic compound extrusion (MATE) efflux pump	51	100
*fosX*	FosX-family fosfomycin resistance hydrolase	51	100
*macB*	MacB ABC-type macrolide efflux transporter	51	100
*bepC*	BepC outer-membrane component of *Brucella* RND efflux system	51	100
*bepD*	BepD periplasmic adaptor of *Brucella* RND efflux system	51	100
*bepE*	BepE inner-membrane permease of *Brucella* RND efflux system	51	100
*bepF*	BepF periplasmic adaptor of *Brucella* RND efflux system	51	100
*bepG*	BepG inner-membrane permease of *Brucella* RND efflux system	51	100

^*a*^ ABC, ATP-binding cassette; MATE, multidrug and toxic compound extrusion; RND, resistance–nodulation–division.

The broader resistance classification recovered 51 resistance-associated loci. Most belonged to the persistent pangenome fraction, including nine antibiotic-resistance-associated loci, 18 multidrug/efflux loci, seven metal-resistance loci**,** five disinfectant/dye-resistance loci, and five other toxic-compound resistance loci. Shell and cloud loci were fewer and lower prevalence: one shell efflux locus in 22 genomes (43.1%), and five cloud loci occurred in 1–11 genomes (2.0–21.6%) (Table 2). More details are provided in Data S4.

**TABLE 2:** Distribution of resistance-associated loci across persistent, shell, and cloud fractions of the *Brucella melitensis* pangenome^*a*^

Class	Primary category	Loci	Median isolate prevalence (%)	Min_isolates	Max_isolates
cloud	Metal resistance	1	7.8	4	4
cloud	Multidrug/efflux AMR	4	7.9	1	11
persistent	Antibiotic resistance	9	100.0	51	51
persistent	Disinfectant/dye resistance	5	100.0	51	51
persistent	Metal resistance	8	100.0	51	51
persistent	Multidrug/efflux AMR	18	100.0	48	51
persistent	Other toxic compound resistance	5	100.0	48	51
shell	Antibiotic resistance	1	45.1	23	23
shell	Multidrug/efflux AMR	1	43.1	22	22

^*a*^ AMR, antimicrobial resistance.

The persistent resistance-associated set included predicted FosX/FosE/FosI-family fosfomycin resistance hydrolase, MacB, NorM, TetB/MFS-type tetracycline-associated transporters, Bcr/CflA-family transporters, RND efflux components, BepC–BepG, SMR/SugE-family biocide efflux proteins, acriflavine-resistance proteins, cadmium-translocating ATPases, copper homeostasis proteins, nickel/cobalt efflux proteins, tellurite-resistance TerB, and fluoride efflux transporters (Fig. 5; Table 3).

**TABLE 3:** Detailed distribution of resistance-associated annotations across persistent, shell, and cloud fractions of the *Brucella melitensis* pangenome^*a*^

Class	Primary category	Annotation	Loci	Isolates detected	Prevalence (%)
cloud	Metal resistance	Copper ABC transporter, permease component (modular protein)	1	4	7.8
cloud	Multidrug/efflux AMR	Bcr/CflA family efflux transporter	1	7	13.7
cloud	Multidrug/efflux AMR	Efflux RND transporter permease subunit	1	1	2.0
cloud	Multidrug/efflux AMR	Multidrug resistance protein a	1	1	2.0
cloud	Multidrug/efflux AMR	Putative multidrug efflux protein	1	11	21.6
persistent	Antibiotic resistance	Beta-lactamase	1	51	100.0
persistent	Antibiotic resistance	Beta-lactamase hydrolase-like protein phosphatase-like domain-containing protein	1	51	100.0
persistent	Antibiotic resistance	Beta-lactamase-like	1	51	100.0
persistent	Antibiotic resistance	FosX/FosE/FosI family fosfomycin resistance hydrolase	1	51	100.0
persistent	Antibiotic resistance	Macrolide export ATP-binding/permease protein MacB	1	51	100.0
persistent	Antibiotic resistance	Tetracycline resistance protein	1	51	100.0
persistent	Antibiotic resistance	Tetracycline resistance protein TetB:Drug resistance transporter EmrB/QacA subfamily:Major facilitator superfamily (MFS)	1	51	100.0
persistent	Antibiotic resistance	Tetracycline resistance protein:NADH-ubiquinone oxidoreductase, chain 4:General substrate transporter:Major facilitator super	1	51	100.0
persistent	Antibiotic resistance	Putative multidrug resistance protein NorM	1	51	100.0
persistent	Disinfectant/dye resistance	Acriflavin resistance protein	2	51	100.0
persistent	Disinfectant/dye resistance	Drug resistance transporter, EmrB/QacA family	1	51	100.0
persistent	Disinfectant/dye resistance	Small multidrug resistance protein	1	51	100.0
persistent	Disinfectant/dye resistance	Quaternary ammonium compound efflux SMR transporter SugE	1	51	100.0
persistent	Metal resistance	Cadmium-translocating P-type ATPase	2	51	100.0
persistent	Metal resistance	Co2+/Mg2 + efflux protein ApaG	1	51	100.0
persistent	Metal resistance	Copper ABC transporter, ATP-binding protein	1	51	100.0
persistent	Metal resistance	Nickel/cobalt efflux system	1	51	100.0
persistent	Metal resistance	Periplasmic regulator RcnB of Ni and Co efflux	1	51	100.0
persistent	Metal resistance	Copper chaperone CopZ	1	51	100.0
persistent	Metal resistance	Multicopper oxidase CueO	1	51	100.0
persistent	Multidrug/efflux AMR	Bcr/CflA family efflux transporter	1	51	100.0
persistent	Multidrug/efflux AMR	Drug resistance transporter, Bcr/CflA family	1	51	100.0
persistent	Multidrug/efflux AMR	Efflux RND transporter periplasmic adaptor subunit	2	51	100.0
persistent	Multidrug/efflux AMR	Efflux RND transporter permease subunit	1	51	100.0
persistent	Multidrug/efflux AMR	Multidrug resistance protein	2	51	100.0
persistent	Multidrug/efflux AMR	Multidrug resistance protein a	1	51	100.0
persistent	Multidrug/efflux AMR	Multidrug resistance protein b	2	51	100.0
persistent	Multidrug/efflux AMR	RND efflux pump membrane fusion protein barrel-sandwich domain-containing protein	3	51	100.0
persistent	Multidrug/efflux AMR	Multidrug efflux RND transporter outer membrane subunit BepC	1	51	100.0
persistent	Multidrug/efflux AMR	Multidrug efflux RND transporter periplasmic adaptor subunit BepD	1	51	100.0
persistent	Multidrug/efflux AMR	Multidrug efflux RND transporter periplasmic adaptor subunit BepF	1	51	100.0
persistent	Multidrug/efflux AMR	Multidrug efflux RND transporter permease subunit BepE	1	51	100.0
persistent	Multidrug/efflux AMR	Multidrug efflux RND transporter permease subunit BepG	1	51	100.0
persistent	Other toxic compound resistance	Tellurite resistance TerB family protein	1	51	100.0
persistent	Other toxic compound resistance	Fluoride efflux transporter CrcB	4	51	100.0
shell	Antibiotic resistance	Beta-lactamase-related domain-containing protein	1	23	45.1
shell	Multidrug/efflux AMR	Efflux pump protein	1	22	43.1

^*a*^ ABC, ATP-binding cassette; AMR, antimicrobial resistance; MFS, major facilitator superfamily; RND, resistance–nodulation–division; SMR, small multidrug resistance.

Therefore, the resistance-associated profile was dominated by conserved efflux, envelope transport, detoxification, and stress-protection functions, with accessory loci limited to a small number of shell and cloud genes.

### Candidate resistance-associated mutations occur mainly in chromosomal drug-target and efflux/stress loci

Missense mutations were identified in priority chromosomal loci: *folA*, *folP*, *gyrA*, *gyrB*, *parC*, *parE*, and *rpoB*. These genes map to antibiotic classes relevant to brucellosis treatment or susceptibility testing: *folA*/*folP* for trimethoprim–sulfonamide-associated pathways, *gyrA*/*gyrB*/*parC*/*parE* for fluoroquinolone targets, and *rpoB* for rifampicin-associated variation (Table 4).

**TABLE 4:** Amino acid substitutions identified in antimicrobial resistance-associated genes across *Brucella melitensis* isolates^*a*^

Isolate	Gene	Nucleotide change(s)	Amino acid substitution(s)
BM1–BM26, BM30–BM37, BM39–BM46	*folA*	c.1072A > G; c.218G > T	p.Arg73Leu; p.Lys358Glu
BM1–BM3, BM6–BM11, BM13–BM22, BM25–BM26, BM34–BM35, BM37, BM46	*parC*	c.800G > A	p.Arg267His
BM1–BM27, BM30–BM47, BM49	*parE*	c.80A > G	p.Asn27Ser
BM8–BM10	*rpoB*	c.208G > C	p.Gly70Arg
BM27, BM41, BM47, BM49	*folA*	c.1072A > G	p.Lys358Glu
BM38, BM43, BM48	*folA*	c.1062A > C; c.883G > A	p.Arg354Ser; p.Val295Met
BM38, BM43, BM48	*folP*	c.631T > C	p.Phe211Leu
BM38, BM43	*gyrA*	c.1795C > G	p.Leu599Val
BM38, BM43	*rpoB*	c.1886C > T	p.Ala629Val
BM48	*rpoB*	c.1886C > T; c.2954C > T	p.Ala629Val; p.Ala985Val
BM50, BM52, BM53	*folA*	c.1072A > G; c.1298A > C	p.Lys358Glu; p.Lys433Thr
BM50, BM52, BM53	*gyrA*	c.1667G > A	p.Arg556His
BM50, BM52, BM53	*gyrB*	c.647C > G	p.Ala216Gly
BM50, BM52, BM53	*parC*	c.427G > A	p.Glu143Lys

^*a*^ Missense mutations with moderate predicted impact.

The most frequent target-gene variants were *folA* p.Lys358Glu in 47/51 genomes, *parE* p.Asn27Ser in 44/51, *folA* p.Arg73Leu in 40/51, and *parC* p.Arg267His in 25/51. Less frequent variants included *rpoB* p.Gly70Arg in three isolates and a distinct cluster comprising three isolates with *folA* p.Arg354Ser, *folA* p.Val295Met, *folP* p.Phe211Leu, *gyrA* p.Leu599Val, and *rpoB* p.Ala629Val. A second small cluster, BM50, BM52, and BM53, carried *gyrA* p.Arg556His, *gyrB* p.Ala216Gly, *parC* p.Glu143Lys, and *folA* p.Lys433Thr (Table 4).

Additional mutations occurred in genes linked to efflux, membrane adaptation, and intrinsic stress tolerance. *bepE* was mutated in 51/51 genomes, with recurrent p.Val936Ala, p.Asn280Asp, and p.Gly870Ser variants; *bepF* p.Thr314Ala occurred in 50/51 genomes; *bepC* p.Val331Leu occurred in 38/51; and *mprF* p.Trp470Cys occurred in 38/51. The *bepCDEFG* system and *mprF* have been reported among efflux or resistance-associated loci in *B. melitensis*, including studies where efflux-gene mutations were present across resistant and susceptible isolates (Data S5).

Overall, the mutation profile was dominated by recurrent variants in conserved chromosomal loci, with smaller isolate clusters carrying additional substitutions in folate-pathway, quinolone-target, rifampicin-target, and efflux-associated genes.

### A secretion-associated protein shows distant homology but remains functionally unresolved

Among the 51 genomes analyzed, a GspF-domain-containing secretion-associated locus was detected only in BM2, a goat (*Capra hircus*) isolate recovered from an abortion-associated vaginal swab. The encoded protein was annotated as a type II secretion system GspF-domain-containing protein.

**Fig 6:**
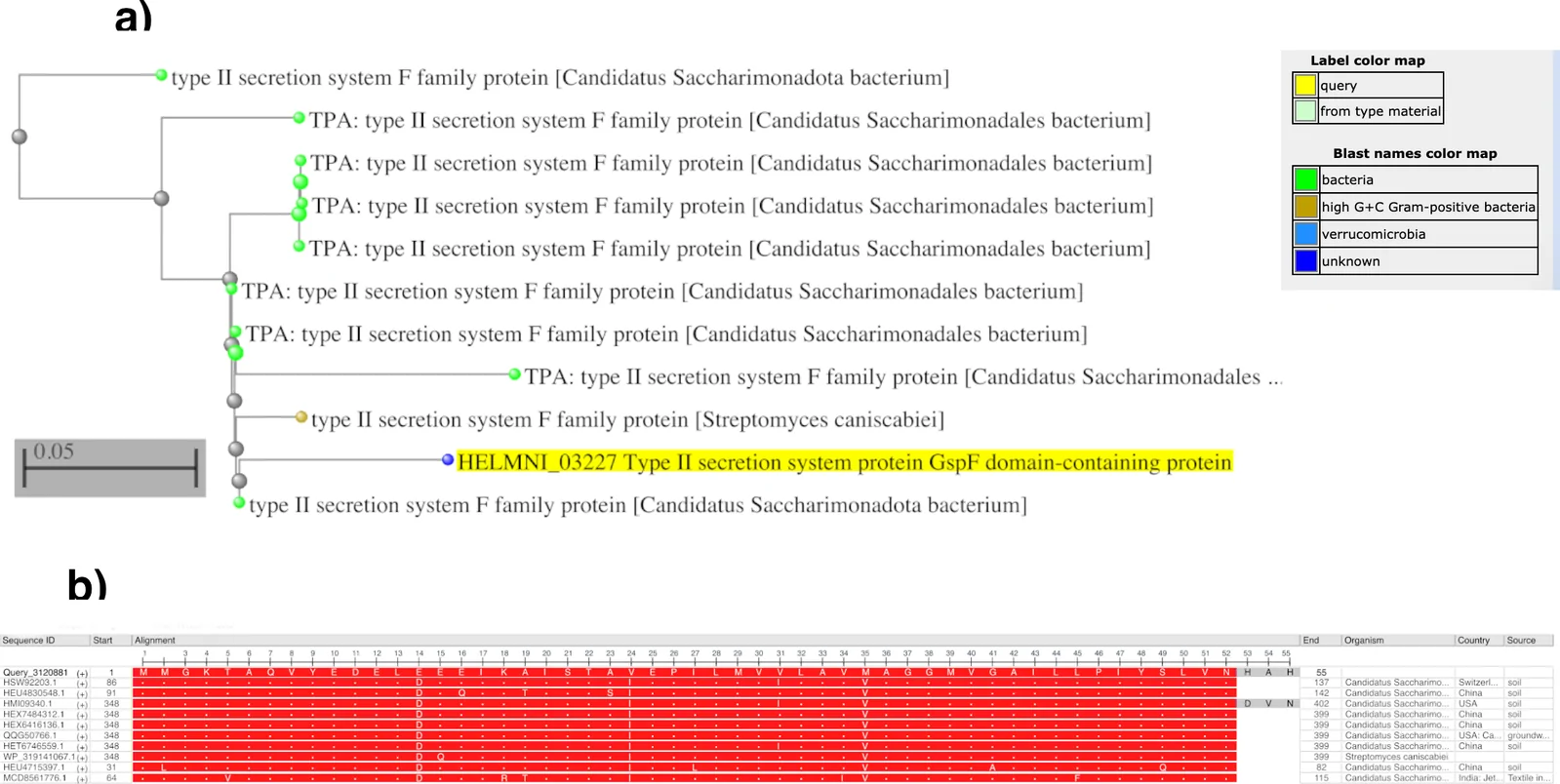
Comparative sequence analysis of the BM2 GspF-domain-containing protein. (**a**) Phylogenetic placement of HELMN1_03227 relative to homologous bacterial proteins annotated as GspF/T2SS-family proteins. The BM2 sequence grouped with homologs from candidate and environmental bacterial taxa, indicating sequence homology of the GspF-domain-containing protein. Branch lengths represent sequence divergence, and node colors indicate taxonomic classification. (**b**) Multiple sequence alignment of HELMN1_03227 and representative homologs showing conserved residues across aligned regions. These similarities support domain-level homology but do not establish the presence of a complete or functional type II secretion system in BM2.

Phylogenetic placement of the BM2 protein placed it among proteins annotated as type II secretion system F family proteins, with the closest labeled neighbors from *Candidatus* Saccharimonadota/Saccharimonadales bacteria and *Streptomyces caniscabiei* (Fig. 6).

GspF is a component of the inner-membrane platform of bacterial type II secretion systems, which in canonical T2SS machinery works with other Gsp proteins during pseudopilus assembly and extracellular protein secretion. In this data set, however, the signal was limited to a single GspF-domain protein in one genome, without evidence of a complete T2SS gene cluster or conserved distribution across the 51-genome panel.

Thus, the BM2 locus is best reported as an isolated, GspF-domain-containing secretion-associated protein with distant homology to T2SS-family proteins.

## DISCUSSION

This study resolves the distribution of genomic diversity across 51 available sub-Saharan African *B. melitensis* genomes. Core-genome phylogeny separated ST12, ST7, ST8, ST42, and a novel sequence type, while pangenome and virulence analyses demonstrated extensive conservation of gene content and intracellular pathogenesis-associated systems. The combined genomic pattern identifies core-SNP lineage structure and localized accessory variation as the principal sources of differentiation within the analyzed regional collection. However, the data set does not constitute a prevalence survey or a complete representation of sub-Saharan African *B. melitensis*. Its geographic composition, with a concentration of genomes from Ethiopia and other countries (Fig. 1), means that subsequent lineage and host-associated patterns are properties of the available curated genomes.

The dominant ST12-associated clade contained most genomes, but core-SNP analysis further separated closely related pairs and subclusters within this background, showing the added resolution of genome-wide SNP analysis over broad sequence-type assignment alone (Fig. 1). Georgi et al. showed that WGS-derived typing can complement epidemiological investigations (5), although metadata often limit fine-scale trace-back inference; similar limitations apply here because host, country, and collection histories were not uniformly sampled. The presence of ST8, ST7, ST42, and a novel sequence type outside the dominant ST12-associated clade indicates that the available sub-Saharan African genomes are not phylogenetically uniform (Fig. 1). This finding extends earlier global phylogeographic work showing that *B. melitensis* contains geographically structured lineages and that additional sampling from Africa is likely to reveal under-characterized diversity (20, 21). However, ST12 is not necessarily the dominant regional lineage across sub-Saharan Africa; rather, it is the dominant lineage within the available curated genome set. The distribution of human, goat, sheep, and cattle isolates across Fig. 1 supports a shared genomic background sampled across host categories, consistent with the zoonotic ecology of brucellosis, but it does not establish direct transmission, source attribution, or direction of spillover. Human brucellosis is commonly linked to contact with infected animals or contaminated animal products, but genomic metadata alone are insufficient to reconstruct specific livestock–human transmission routes (3, 22). Thus, the phylogeny places sub-Saharan African *B. melitensis* genomes into a genome-resolved population framework while also showing that stronger regional inference will require balanced sampling, matched epidemiological metadata, and denser representation from livestock and human surveillance systems.

Pangenome analysis quantified the contribution of gene-content variation to genomic diversity in the regional collection (Fig. 2). The 3,049 persistent families, together with the small shell and cloud fractions, demonstrate extensive conservation of gene content and limited accessory expansion across the 51 genomes. This fits the broader biology of *Brucella* as a host-adapted, facultative intracellular genus with relatively conserved genome organization (Fig. 3), but it also refines that understanding for the currently available African genomes by showing that regional diversity is not mainly expressed through large accessory-genome turnover. Recent *B. melitensis* analyses from other settings have similarly reported large core-genome fractions; for example, a 2025 genomic study of human *B. melitensis* isolates from southern Tunisia reported 3,054 core genes among 3,304 pangenome genes, supporting the view that many *B. melitensis* populations retain a strongly conserved gene backbone (23). At the same time, wider genus-level analyses show that *Brucella* can display an open pangenome when more species and ecological backgrounds are included, indicating that accessory diversity becomes more visible at broader taxonomic or ecological scales (24, 25). The limited number of RGPs, spots, and cloud-only modules in this data set therefore suggests that, within these 51 genomes, biologically relevant variation is likely concentrated in restricted accessory regions rather than dispersed across the genome. For surveillance, gene presence–absence alone may underestimate meaningful diversity in *B. melitensis* and should be used together with core-SNP structure, allelic variation, mobile-element context, and phenotype-linked data.

Virulence-gene profiling showed that the annotated intracellular pathogenic repertoire is highly conserved across the available sub-Saharan African *B. melitensis* genomes (Fig. 4). This finding is biologically coherent with the established pathogenesis of *Brucella*, in which disease depends less on classical exotoxin production and more on intracellular entry, controlled trafficking of the *Brucella*-containing vacuole, immune modulation, resistance to host stress, and persistence within phagocytic cells. The strongest signal in this data set was the conservation of systems linked to immune modulation and effector delivery, including the VirB type IV secretion system, which is widely recognized as a central determinant of *Brucella* intracellular survival and host-cell manipulation (7). The conservation of LPS/O-antigen, cyclic β-1,2-glucan, urease/acid-resistance, oxidative and nitrosative stress, respiratory survival, and DNA-repair-associated functions further supports a model in which the genomes share the core molecular machinery needed for intracellular adaptation rather than showing major virulence-gene turnover (Fig. 4). The limited variability of *virB10* and *bmaC* represents minor gene-level variation within an otherwise conserved virulence architecture. The *bmaC* and related *Brucella* adhesins have been linked to host-cell adhesion, whereas VirB components are associated with intracellular survival after entry; the two variable genes therefore fall within biologically relevant but functionally distinct stages of infection (6). However, the analysis measures gene presence and annotation, not expression, regulation, secretion activity, or infection phenotype. Thus, the study shows that the African *B. melitensis* genomes retain a conserved virulence-gene backbone while leaving open whether allelic variation, gene regulation, or host-specific interactions alter virulence among isolates.

The MGE analysis identifies localized genomic plasticity within an otherwise conserved *B. melitensis* background (Fig. 5). Most genomes carried compact MGE-associated profiles, whereas a small number of isolates showed expanded MGE content, indicating that mobile-associated variation is unevenly distributed rather than a uniform feature of the data set. This pattern is consistent with broader *Brucella* genomic studies showing that classical intracellular species are relatively conserved but still retain mobile-element-associated signals that can contribute to genome plasticity, lineage differentiation, and localized accessory variation (26, 27). In particular, IS711 is a well-recognized *Brucella*-associated insertion sequence, and its genomic distribution has been linked to *Brucella* evolution, plasticity, and host-associated genomic patterns. The predominance of transposases, insertion-sequence-associated features, and hypothetical proteins in this data set therefore supports a model in which MGE-associated regions mark focal sites of genome variability (Data S3). The restricted occurrence of passenger genes (Fig. 5), including loci linked to membrane transport, polysaccharide biosynthesis, stress adaptation, and metabolism, suggests that some mobile-associated regions may carry functional cargo; however, their biological effects cannot be inferred from annotation alone. Comparative *Brucella* studies have shown that accessory regions often contain mobile elements, phage-related genes, integrases, and recombination-associated functions, but the presence of such features does not necessarily establish active transfer, altered virulence, or host adaptation (25). Thus, the MGE signal in this study refines the pangenome result: the available sub-Saharan African *B. melitensis* genomes are not highly accessory-genome-driven but contain discrete mobile-associated regions that may provide a limited substrate for lineage-specific variation.

The resistance-associated annotation profile was dominated by conserved efflux, detoxification, envelope-transport, metal-homeostasis, and toxic-compound response functions rather than by a diverse accessory resistome (Tables 1 and 2). The universal detection of *norM, fosX, macB*, and the *bepCDEFG* RND efflux system suggests that these loci form part of the shared physiological repertoire of the sampled *B. melitensis* genomes (Table 1). This is consistent with previous work showing that *Brucella* efflux systems can contribute to antimicrobial tolerance and intracellular fitness while also emphasizing that their exact contribution to resistance phenotypes remains incompletely resolved (28). More recent *B. melitensis* studies have also highlighted efflux-associated loci as recurring components of resistance-focused genomic analyses, while noting that the mechanistic roles of these pumps require further phenotypic validation (8, 29). In the present data set, the predominance of persistent resistance-associated annotations therefore points to intrinsic stress-response and transport capacity rather than widespread acquired antimicrobial resistance (Table 2). The annotation terms such as “resistance protein,” “efflux transporter,” or “beta-lactamase-like” do not by themselves establish clinical resistance, particularly in the absence of matched antimicrobial susceptibility testing. The small number of shell and cloud resistance-associated loci indicates that accessory resistance-related content exists but contributes only a limited layer of variation compared with the conserved efflux and detoxification background (Table 2). Thus, this section identifies candidate intrinsic tolerance and stress-response systems that should be interpreted together with MIC data, expression studies, and mutation-level analyses rather than as direct evidence of resistant *B. melitensis* isolates.

Mutation screening refined the resistance-associated annotation profile by identifying recurrent allelic variation in chromosomal drug-target, folate-pathway, efflux, and envelope/stress-associated loci (Data S5). The detection of variants in genes corresponds to antimicrobial classes used in brucellosis treatment or susceptibility evaluation, including rifampicin, fluoroquinolones, and trimethoprim–sulfamethoxazole. Previous studies have similarly used *rpoB* and quinolone-target genes to screen for rifampicin- and fluoroquinolone-associated variation, but they also show that genotype–phenotype interpretation is not straightforward and that some recurrent variants may reflect lineage background (30, 31). In the present data set, the high-frequency variants, including *folA* p.Lys358Glu, *parE* p.Asn27Ser, *folA* p.Arg73Leu, and *parC* p.Arg267His (Table 3), are recurrent candidate alleles in conserved chromosomal loci, not as validated resistance determinants. The *rpoB* variation can be mechanistically linked to resistance, but the resistance effect depends on the specific substitution and phenotypic context (32, 33). The additional variation in *bepC*, *bepE*, *bepF*, and *mprF* extends the signal from drug-target genes to efflux, membrane adaptation, and intrinsic stress-tolerance systems (Data S5). Similar recent genomic work has reported *bepCDEFG* and *mprF* as widely distributed resistance-associated or tolerance-associated loci in *B. melitensis*, supporting their relevance as candidates for phenotype-linked follow-up rather than direct resistance markers (23). Thus, the main value of this analysis is prioritization: it identifies specific alleles and isolates clusters for future MIC-linked testing, expression analysis, and functional validation.

The BM2 secretion-associated locus adds a rare accessory signal to the otherwise VirB-centered secretion architecture of the data set. GspF is a component of the type II secretion system inner-membrane assembly platform; in canonical T2SS machinery, it functions with other Gsp proteins, including GspE, GspL, and GspM, during the assembly of the pseudopilus-associated secretion complex (34, 35). Therefore, the detection of a single GspF-domain-containing protein is not used as evidence for a complete or functional T2SS in *B. melitensis*. The established *Brucella* secretion biology is dominated by the VirB type IV secretion system, which mediates intracellular survival, *Brucella*-containing vacuole trafficking, host immune modulation, and effector delivery (7, 36). The distant homology of the BM2 protein to T2SS-family proteins from phylogenetically distant bacteria suggests conservation of a secretion-associated domain outside the core *Brucella* virulence repertoire, rather than a dataset-wide secretion-system pattern (Fig. 6). Thus, the BM2 locus is a localized accessory variation that broadens the observed genomic diversity of the data set.

### Conclusion

This study provides a genome-resolved analysis of available sub-Saharan African *B. melitensis* genomes from human and animal hosts. The analyzed collection was characterized by a highly conserved core genome, a dominant ST12-associated lineage, near-complete conservation of intracellular virulence determinants, and limited accessory-genome expansion. Genomic variability was concentrated within small mobile-associated regions and chromosomal mutations occurring in AMR-relevant loci, including genes linked to rifampicin, fluoroquinolone, and folate-pathway susceptibility. However, the detected mutations did not correspond to validated resistance-conferring alleles and therefore represent candidate genomic variation requiring phenotypic confirmation.

Together, these findings show that the available regional genomes are genetically conserved but phylogenetically structured, with diversification concentrated in core-genome SNPs and localized genomic plasticity. The study establishes a regional comparative-genomics framework for zoonotic genomic surveillance and provides a foundation for future phenotype-linked investigation of virulence, transmission, and antimicrobial susceptibility in *B. melitensis*.

## Data Availability

All raw sequencing reads and genome assemblies analyzed in this study are publicly available through the National Center for Biotechnology Information (NCBI). The corresponding accession numbers, BioProject and BioSample identifiers, host information, collection metadata, and genome-quality details are provided in Data S1.

## AFTER EPUB

*[This article was published on 19 August 2026 with formatting errors. The errors were corrected in the current version, posted on 21 September 2026.]*

## SUPPLEMENTAL MATERIAL

Data S1 spectrum.01703-26-s0001.xlsx Genome dataset metadata, quality-control metrics, assembly statistics, species confirmation, and sequence typing results for the curated *Brucella melitensis* genome panel. This workbook summarizes raw-read accession metadata, fastp read-quality metrics, QUAST assembly statistics, species-confirmation outputs, and MLST profiles used to curate and define the sub-Saharan African *B. melitensis* dataset analyzed in this study.

Data S2 spectrum.01703-26-s0002.xlsx Virulence-factor annotations and functional module distribution across the *Brucella melitensis* genome panel. This workbook provides VFDB-derived virulence-gene calls for each isolate, genome-level virulence-gene recovery, gene-frequency summaries, variably detected virulence genes, annotated virulence protein functions, and distribution of virulence-associated functional modules.

Data S3 spectrum.01703-26-s0003.xlsx Mobile genetic element-associated annotations and passenger-gene content across *Brucella melitensis* isolates. This workbook contains Bakta-derived annotations of mobile genetic element-associated regions, including per-isolate MGE feature counts, MGE class summaries, passenger-gene-containing elements, mobility-associated genes, and passenger-associated genes identified across the dataset.

Data S4 spectrum.01703-26-s0004.xlsx Curated resistance-associated loci across persistent, shell, and cloud pangenome fractions. This workbook presents literature-guided classification of resistance-related annotations, including antibiotic resistance, multidrug/efflux systems, metal resistance, disinfectant/dye resistance, and toxic-compound resistance loci, together with prevalence, evidence strength, pangenome partition, and supporting reference information.

Data S5 spectrum.01703-26-s0005.xlsx Amino acid substitutions identified in antimicrobial resistance-associated and efflux/stress-related genes. This workbook lists moderate-impact missense variants detected in priority AMR-associated chromosomal loci, including folate-pathway, quinolone-target, rifampicin-associated, efflux, membrane-adaptation, and stress-related genes across the analyzed *B. melitensis* isolates.

Fig. S1 spectrum.01703-26-s0006.pdf Virulence and mobile-element feature distribution across 51 sub-Saharan African *Brucella melitensis* genomes.

ASM does not own the copyrights to Supplemental Material that may be linked to, or accessed through, an article. The authors have granted ASM a non-exclusive, world-wide license to publish the Supplemental Material files. Please contact the corresponding author directly for reuse.
