# Supplementary material for: Conserved intracellular virulence architecture and focal genomic diversification in sub-Saharan African Brucella melitensis
Source: Microbiol Spectr. 2026 Aug 19;14(10):e01703-26. doi: 10.1128/spectrum.01703-26 (PMC13689090; doi:10.1128/spectrum.01703-26)
Supplement: Supplementary file 1 [file spectrum.01703-26-s0006.pdf]

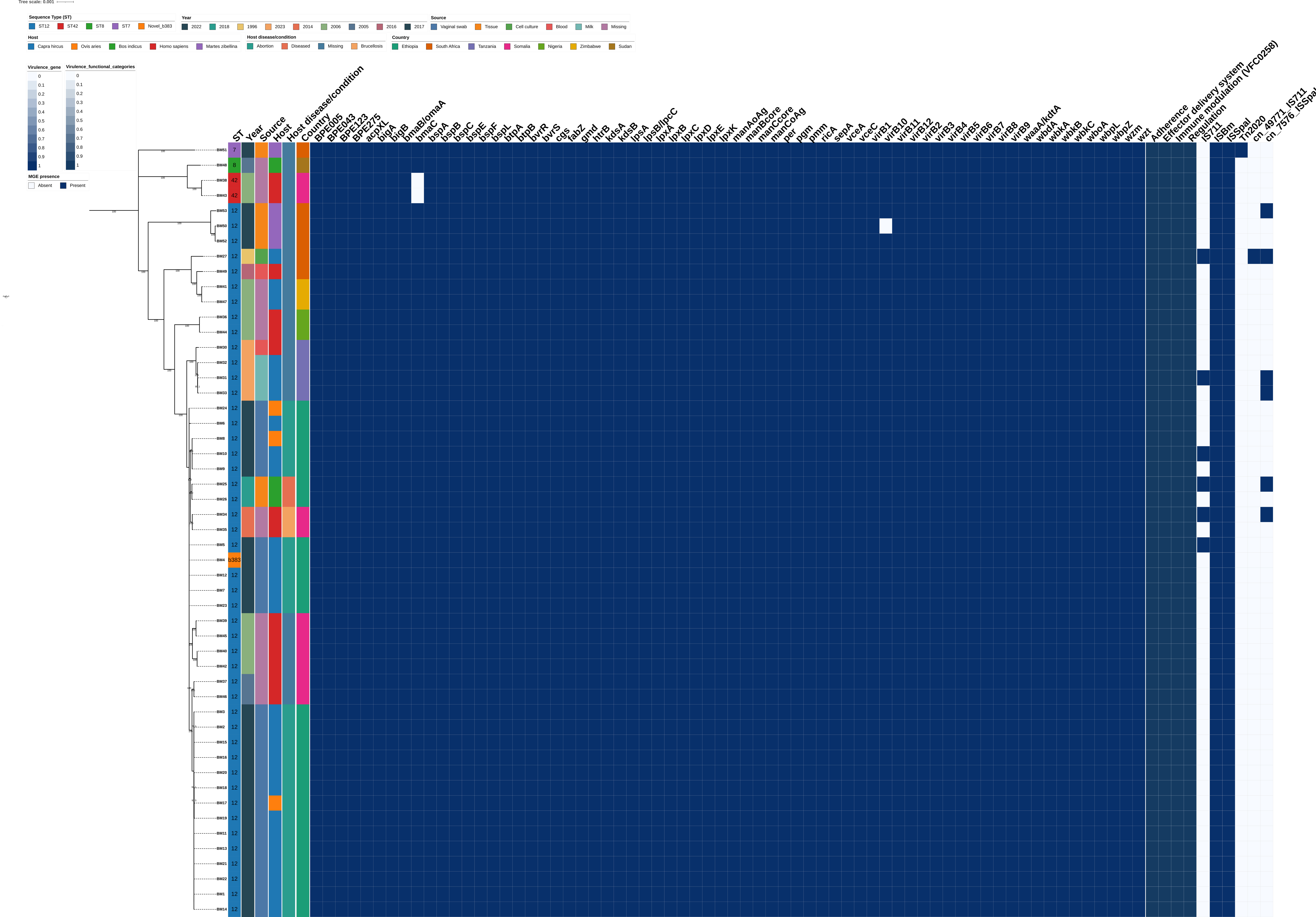

Tree scale: 0.001
Sequence Type (ST)
ST12
ST42
ST8
ST7
Novel_b383
Year
2022
2018
1996
2023
2014
2006
2005
2016
2017
Source
Vaginal swab
Tissue
Cell culture
Blood
Milk
Missing
Host
Capra hircus
Ovis aries
Bos indicus
Homo sapiens
Martes zibellina
Host disease/condition
Abortion
Diseased
Missing
Brucellosis
Country
Ethiopia
South Africa
Tanzania
Somalia
Nigeria
Zimbabwe
Sudan
Virulence_gene
Virulence_functional_categories
MGE presence
Absent
Present
ST
Year
Source
Host
Host disease/condition
Country
BPE005
BPE043
BPE123
BPE275
acpXL
bigA
bigB
bmaB/omaA
bmaC
bspA
bspB
bspC
bspE
bspF
bspL
btpA
btpB
bvrR
bvrS
cgs
fabZ
gmd
htrB
kdsA
kdsB
lpsA
lpsB/lpcC
lpxA
lpxB
lpxC
lpxD
lpxE
lpxK
manAoAg
manBcore
manCcore
manCoAg
per
pgm
pmm
ricA
sepA
vceA
vceC
virB1
virB10
virB11
virB12
virB2
virB3
virB4
virB5
virB6
virB7
virB8
virB9
waaA/kdtA
wbdA
wbkA
wbkB
wbkC
wboA
wbpL
wbpZ
wzm
wzt
Adherence
Effector delivery system
Immune modulation (VFC0258)
Regulation
IS711
ISBm
ISSpal
Tn2020
cn_49771_IS711
cn_7576_ISSpal
BM51
BM48
BM38
BM43
BM53
BM50
BM52
BM27
BM49
BM41
BM47
BM36
BM44
BM30
BM32
BM31
BM33
BM24
BM6
BM8
BM10
BM9
BM25
BM26
BM34
BM35
BM5
BM4
BM12
BM7
BM23
BM39
BM45
BM40
BM42
BM37
BM46
BM3
BM2
BM15
BM16
BM20
BM18
BM17
BM19
BM11
BM13
BM21
BM22
BM1
BM14

SUPPLEMENTARY FIGURE LEGEND

Conserved intracellular virulence architecture and focal genomic diversification in sub-Saharan African Brucella melitensis

Supplementary Figure S1. Complete virulence-gene presence–absence profile across 51 sub-Saharan African Brucella melitensis genomes. Heatmap showing the distribution of 66 Virulence Factor Database (VFDB)-associated genes across the curated genome panel. Rows represent virulence genes and columns represent individual genomes; cells indicate whether each gene was detected in each genome. Associated annotation tracks summarize available isolate metadata and virulence-module classification. The detected repertoire comprises genes assigned to immune modulation, effector delivery systems, adherence, and regulation. Forty-eight genomes contained all 66 genes, whereas three genomes contained 65 genes (98.48% of the detected repertoire). The only variably detected genes were virB10, present in 50 of 51 genomes, and bmaC, present in 49 of 51 genomes.
